# Risk factors for nasopharyngeal carriage of drug-resistant *Streptococcus pneumoniae*: data from a nation-wide surveillance study in Greece

**DOI:** 10.1186/1471-2334-9-120

**Published:** 2009-07-29

**Authors:** Ioannis Katsarolis, Garyphallia Poulakou, Antonios Analitis, Irini Matthaiopoulou, Emmanuel Roilides, Charalampos Antachopoulos, Dimitrios A Kafetzis, Georgios L Daikos, Regina Vorou, Christina Koubaniou, Ioannis Pneumatikos, Georgios Samonis, Vasiliki Syriopoulou, Helen Giamarellou, Kyriaki Kanellakopoulou

**Affiliations:** 1 4th Dept of Internal Medicine, Athens Medical School, ATTIKON University General Hospital, Athens Greece; 2 Department of Hygiene and Epidemiology, Athens Medical School, Athens Greece; 3 3rd Dept of Pediatrics, Aristotle University of Thessaloniki, Hippokration Hospital, Thessaloniki, Greece; 4 2nd Dept of Pediatrics, Athens Medical School, P. & A. Kyriakou Children's Hospital, Athens Greece; 5 1st Dept of Propaedeutic Medicine, Athens Medical School, Laiko General Hospital, Athens Greece; 6 Pneumonology Department, University of Ioannina Medical School, Ioannina Greece; 7 Alexandroupolis University Hospital, Democritus University of Thrace Medical School, Alexandroupolis Greece; 8 Department of Internal Medicine, University Hospital of Herakleion, Herakleion Crete, Greece; 9 1st Department of Pediatrics, Athens Medical School, Aghia Sophia Children's Hospital, Athens Greece

## Abstract

**Background:**

A nation-wide surveillance study was conducted in Greece in order to provide a representative depiction of pneumococcal carriage in the pre-vaccination era and to evaluate potential risk factors for carriage of resistant strains in healthy preschool children attending daycare centers.

**Methods:**

A study group was organized with the responsibility to collect nasopharyngeal samples from children. Questionnaires provided demographic data, data on antibiotic consumption, family and household data, and medical history data. Pneumococcal isolates were tested for their susceptibility to various antimicrobial agents and resistant strains were serotyped.

**Results:**

Between February and May 2004, from a total population of 2536 healthy children, a yield of 746 pneumococci was isolated (carriage rate 29.41%). Resistance rates differed among geographic regions. Recent antibiotic use in the last month was strongly associated with the isolation of resistant pneumococci to a single or multiple antibiotics. Serotypes 19F, 14, 9V, 23F and 6B formed 70.6% of the total number of resistant strains serotyped.

**Conclusion:**

Recent antibiotic use is a significant risk factor for the colonization of otherwise healthy children's nasopharynx by resistant strains of *S pneumoniae*. The heptavalent pneumococcal conjugate vaccine could provide coverage for a significant proportion of resistant strains in the Greek community. A combined strategy of vaccination and prudent antibiotic use could provide a means for combating pneumococcal resistance.

## Background

*Streptococcus pneumoniae *is the most commonly isolated pathogen in community-acquired respiratory tract infections, being the causative agent of a variety of infections, such as acute otitis media, sinusitis and pneumonia [1]. The development of antibiotic resistance in *S pneumoniae *over the last two decades has raised a global concern [2], an evolution generally attributed to an extensive consumption of antibiotics [3].

The nasopharynx of preschool children is the ecological niche of *S pneumoniae *[4]. Nasopharyngeal colonization is a prerequisite for progression to pneumococcal disease and an important source of horizontal spread in the community, especially in settings with high crowding index [1]. Antibiotic-resistant pneumococci involve mostly only a few serogroups in the majority of developed countries (6, 9, 14, 19 and 23). The routine immunization with the heptavalent pneumococcal conjugate vaccine has been shown to decrease the incidence of vaccine-type antibiotic-resistant pneumococci both in invasive diseases; nasopharyngeal colonization was also dramatically decreased in vaccinated individuals and their contacts [5].

In Greece, surveillance studies addressing the issue of resistance and serotype distribution in nasopharyngeal isolates of *Streptococcus pneumoniae *have been conducted in recent years [6,7]. Although these studies covered specific regions of the Greek territory, a rise in the frequency of antibiotic-resistant pneumococci has been documented. Recently, another study [8] assessed at regional level the impact of the heptavalent pneumococcal vaccination on the nasopharyngeal carriage of penicillin-resistant pneumococci among day-care attendees.

The heptavalent pneumococcal conjugate vaccine became available in October 2004 and formed part of the national immunization schedule in January 2006. Between February and May 2004 (pre-vaccination era), the present large-scale study was conducted in Greece, through a multi-center initiative task force, in order to provide for the first time a nation-wide representative depiction of carriage of pneumococcal strains isolated from the nasopharynx of healthy preschool children, attending daycare centers and kindergartens. Data on resistance rates and serotype distribution at nation-wide level from this study were published in correlation with data for clinical strains from children and adults for the same period of time [9]. The purpose of the present article is to identify and evaluate risk factors for carriage of resistant pneumococcal strains in this specific population in the pre-vaccination era.

## Methods

### Study group and study population

A study group of 12 hospitals was organized under the coordination of the 4^th ^Department of Internal Medicine and the Infectious Diseases Laboratory of Athens University Medical School (see Acknowledgements section). The research protocol was approved by the Ministries of Health and Education and the Ethics Committees of all cooperating hospitals. A network was formed consisting of local teams with the responsibility to inform parents and collect appropriately filled questionnaires, collect and culture nasopharyngeal samples from the children, isolate presumed pneumococcal strains, store and ship isolated strains to the central laboratory (Laboratory for Infectious Diseases and Antimicrobial Chemotherapy, 4^th ^Dept of Internal Medicine, Athens University School of Medicine, University General Hospital ATTIKON). The single central laboratory undertook the microbiological procedures and the input of data from the questionnaires in a purpose-structured database.

Questionnaires were distributed to the parents by the local teams of doctors visiting the day-care centres 1–2 days before the sampling visit. On the sampling day, questionnaires were reviewed by the doctors' team. In the section of recent antibiotic use, parents were asked to provide generic or commercial names of all medications received in the last month or trimester and the appropriate classification was performed by the responsible doctors in the sampling site. Questionnaires were signed by the parents, were anonymous but labeled to match the respective sample per child, and consisted of three parts: a) demographic data, i.e. age, gender, name of daycare center, area and prefecture; b) data on antibiotic consumption in the last month and in the last trimester preceding the sampling, including: number of antibiotic courses, name of antimicrobial and reason for its prescription, mode of supply (over-the-counter or by prescription), c) family and household data, i.e. parents working in health care, number of siblings <8 years of age, and d) co morbidities and past medical history data: admission to hospital in the last year, history of « respiratory tract infections », otitis media history in the previous year and steroid use. Children without signed questionnaires by the parents were excluded from sampling, as well as those receiving antibiotics for active infection. Samples without appropriately filled questionnaires were microbiologically processed but were excluded from risk factor analysis.

### Sampling procedure and microbiological process of pneumococcal strains

The period of sampling started February 2004 and lasted until the end of May 2004. Carriage samples were collected per nasally with sterile swabs on flexible aluminium wire (Medical Wire & Equipment, Corsham, UK). Swabs were immediately plated on Columbia agar plates (Becton Dickinson, Sparks, MD) supplemented with 5% defibrinated horse blood and packed in 5% CO2 Gas Pak Pouches (Becton Dickinson).

Streptococcus pneumoniae isolates were identified by standard microbiological procedures. Susceptibility testing was performed with the E-test methodology (AB Biodisk, Solna, Sweden) for penicillin(PG), cefuroxime (CXM), ceftriaxone(CTX), erythromycin(ERY), trimethoprim/sulfamethoxazole (COT), tetracycline (TET), moxifloxacin, levofloxacin and ciprofloxacin (CIP). Strains displaying resistance were serotyped by the Quellung reaction using the 12 pooled antisera Pneumotest panel and selected factor sera (Statens Serum Institut, Copenhagen, Denmark). Methodology of sampling, isolation, identification and serotyping of pneumococcal strains, as well as susceptibility testing is mentioned elsewhere in detail [9].

### Definitions of terms

Clinical and Laboratory Standards Institute methodology and breakpoints for *non-susceptibility *and *resistance *were used for all antibiotics [10], except for ciprofloxacin. For ciprofloxacin, the epidemiological cut-off of 2 mg/L was used as an indicator of resistance, according to the European Committee on Antimicrobial Susceptibility Testing (EUCAST) 2006 recommendations [11]. Strains with an MIC value higher than the susceptibility breakpoint were characterized as *"non-susceptible"*, i.e. intermediate and fully resistant strains. *Combined resistance *was defined as resistance to two different classes of antibiotics (e.g. penicillin and erythromycin) irrespective of other classes. *Multiple resistance *(or *multi-resistance*) was defined as resistance to 3 or more antibiotics of different classes [2].

### Statistical analysis

Potential risk factors for carriage of pneumococci and in particular of resistant pneumococcal strains were identified by univariate analysis. Antibiotic use was further analyzed using multivariate logistic regression analysis, controlling for variables that were significant in the univariate analysis and those that could correlate with an increased risk of carriage of resistant/non-susceptible strains from a clinical point of view. The following parameters were entered for analysis in the multivariate logistic regression models: age, gender, location of day care center or kindergarten, number of young siblings in the household with age <8 years, hospitalization, recent use of antibiotic by subject, steroid use, history of otitis media and history of respiratory tract infections. A *P *value of = 0.05 was considered to be statistically significant.

## Results

### Study population and demographics

**Figure 1:**
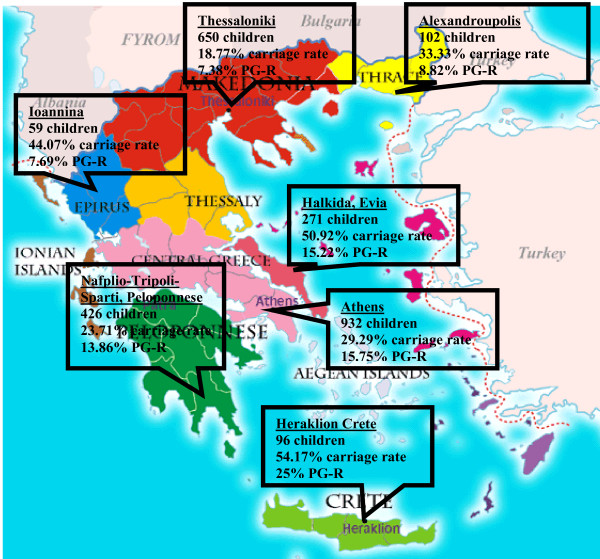
**Map of Greece with the geographic location of different regions participating in the 2004 *Streptococcus pneumoniae *nasopharyngeal study For every region, the number of children sampled, the respective carriage rates and penicillin resistance rates (PGR%) are provided in the boxes**.

Between February and May 2004, consecutive nasopharyngeal swabs and questionnaires were collected from 2595 healthy children (median age ± S.D. 4.3 ± 1.1 years, male: female ratio 1:1), of which, after careful evaluation of the accompanying questionnaires, 2536 were included in the study analysis (2536/2595, i.e. 97.7% of original population). In Table 1, distribution of nasopharyngeal sampling specimens and isolated strains per geographic region is shown. Carriage rates differed significantly among differed regions. In Figure 1, a map of Greece is provided indicating the geographical location of the sampling sites, with the respective sampling population, carriage rates and penicillin resistance rates.

**Table 1:** Distribution of nasopharyngeal samples and carriage rates of pneumococcal strains per geographic region.

City/Prefecture	Total Number of children sampled	Number of pneumococcal strains isolated	Carriage rate %
Athens. Attika	932	273	29.29%

Halkida. Evia	271	138	50.92%*

Thessaloniki. Central Macedonia	650	122	18.77%*

Ioannina. Epirus	59	26	44.07%*

Alexandroupolis. Thrace	102	34	33.33%

Herakleion. Crete	96	52	54.17%*

Nafplio-Tripoli-Sparti. Peloponnese	426	101	23.71%

**Greece in total**	***2536***	***746***	***29.42%***

* flags statistically significant differences at *p *level of = 0.05. as compared to Attika prefecture

### Nasopharyngeal carriage of S pneumoniae strains

A total of 793 pneumococcal strains was isolated in the study from the original 2595 nasopharyngeal specimens (mean carriage rate within study period 793/2595 = 30.55%). Of those, 780 isolates survived shipment and storage and were microbiologically processed, and finally 746 strains were included in the present protocol analysis of the 2536 properly filled questionnaires (mean carriage in per protocol analysis 746/2536 = 29.41%). Carriage of pneumococcal strains was related to age, period and region of sampling, use of antibiotics in the last trimester (last month not included) and history of respiratory infections in the last year. Antibiotic use during the last month did not affect carriage rates of pneumococci. In Table 2, analysis of potential risk factors for nasopharyngeal carriage of pneumococci, as defined by the protocol questionnaire, is listed.

**Table 2:** Risk factors for carriage of pneumococcal strain.

	Odds Ratio (OR)	*P*	95% CI
Gender (boys)	1.019	0.827	0.855–1.211
Age (<3 years old)	0.886	**0.001**	0.824–0.953
Private vs. public daycare centre	0.862	0.128	0.712–1.043
Antibiotic consumption			
In the last month	0.866	0.139	0.717–1.040
In the last trimester	1.262	**0.009**	1.060–1.501
Siblings <8 ys in the family (yes or no)	1.036	0.535	0.926–1.159
History of respiratory tract infections	0.745	**0.024**	0.577–0.962
History of acute otitis media episodes	1.098	0.288	0.923–1.306
Steroid use	1.074	0.525	0.861–1.340
History of hospital admission	0.751	0.296	0.439–1.285
Parents health-care workers	1.176	0.210	0.912–1.517

### Susceptibility testing

In Table 3, results for resistance rates and MIC distribution parameters for all antibiotics tested are shown per region of sampling. Resistance rates differed significantly among different geographic regions. Multiresistant strains formed 23.86% of the carriage isolates (178/746), while combined resistance to penicillin and erythromycin was documented for 75/746 of the total sample (10.05%). No resistance to moxifloxacin and levofloxacin was documented.

**Table 3:** Distribution of MIC50/MIC90 values and resistance/nonsusceptibility rates per geographic region

City/Prefecture	# strains		PG	CXM	CTX	ERY	COT	TET	CIP
Athens.		**%ns-%r**	37.9–15.8	29.8–28.7	1.8–0.4	33.2–32.1	43.6–26	24.2–23.4	3.8
Attika	273	**MIC50/MIC90**	0.023–2	0.047–4	0.023–0.75	0.125–256	0.5–32	0.125–24	0.75–1.5

Halkida.		**%ns-%r**	37–15.2	23.9–21.7	1.4–0	39.1–37.7	51.8–29.2	34.8–30.4	3.8
Evia	138	**MIC50/MIC90**	0.023–2	0.032–3	0.023–0.75	0.125–256	0.75–32	0.19–32	0.75–2

Thessaloniki.		**%ns-%r**	32.8–7.4*	21.3–18	0.8–0	34.2–34.2	38–29.8	21.3–20.5	3.3
Central Macedonia	122	**MIC50/MIC90**	0.032–1	0.032–2	0.016–0.5	0.125–24	0.5–32	0.25–48	0.75–1.5

Ioannina.		**%ns-%r**	19.2*-7.7*	11.5–11.5	2.9–0	42.3–38.5	42.3–19.2	19.2–19.2	0
Epirus	26	**MIC50/MIC90**	0.032–1	0.047–3	0.023–0.38	0.25–96	0.5–18	0.125–24	0.5–1

Alexandroupolis.		**%ns-%r**	11.8*-8.8*	8.8–8.8	0–0	17.6*-17.6	35.3–2.9	14.7–11.8	11.8
Thrace	34	**MIC50/MIC90**	0.023–1	0.023–2	0.023–0.5	0.125–2.5	0.38–2	0.125–24	1–3

Herakleion.		**%ns-%r**	53.8*-25.5*	40.4–34.6	0–0	66.7*-66.7	65.4–26.9	48.1–46.2	6.1
Crete	52	**MIC50/MIC90**	0.12–2	0.5–6	0.12–0.75	32–256	1–32	1.5–96	0.75–1.5

Nafplio-Tripoli-Sparti.		**%ns-%r**	28.0*-14.0*	24.0–24.0	1.0–0.0	14.0*-14.0	30.0–20.0	21.0–21.0	2.0
Peloponnese	101	**MIC50/MIC90**	0.023–2	0.023–2	0.023–0.75	0.094–128	1–2	0.125–24	0.75–2

**Greece in total**	***746***	**%ns-%r**	*34.8–14.1*	*25.7–23.9*	*1.3–0.1*	*33.8–33*	*43.8–25.2*	*26.3–24.8*	*4.0*
		**MIC50/MIC90**	*0.023–1.5*	*0.032–3*	*0.023–0.75*	*0.125–256*	*0.5–32*	*0.125–24*	*0.75–2*

No resistance was recorded for levo- and moxi-floxacin. Respective MIC50/90 values were 0.75–1 and 0.125–0.19 mg/L

PG: penicillin. CXM: cefuroxime. CTX: ceftriaxone. ERY: erythromycin. COT: cotrimoxazole. TET: tetracycline. CIP: ciprofloxacin

*flags statistically significant differences at *p *level of = 0.05 when compared to Attika region

### Risk factors for carriage of resistant pneumococci

Recent antibiotic use in the last month-irrespective of antibiotic class, duration of administration and number of courses of antibiotics- was strongly associated with the isolation of resistant pneumococci to a single or multiple antibiotics. Moreover, increasing number of antibiotic courses in the last month increased the likelihood of isolating a resistant pneumococcal strain Odds Ratio (OR) per antibiotic course for penicillin resistance = 1.35, 95% Confidence Interval (CI) <0.99–1.83> *P *= 0.05, and OR for erythromycin resistance = 1.43, CI <1.11–1.83> *P *= 0.004). Combined resistance to penicillin and erythromycin and multiresistance (3 or more classes of antibiotics) were also strongly related to antibiotic use in the month preceding sampling (OR for combined resistance = 2.09, CI <1.24–3.52> *P *= 0.002, and OR for multiresistance = 2.02, CI <1.38–2.96> *P *< 0.001). In terms of non-susceptibility (i.e. intermediate and fully resistant strains together), antibiotic use both in the last month and in the preceding 3 months (last month excluded) held a statistically significant correlation with carriage of a penicillin and/or erythromycin non susceptible strain. For children that had received an antibiotic course in the last trimester, the possibility of carrying a resistant (or a non-susceptible) strain in their nasopharynx was even greater, if they had additionally received an antibiotic course in the last month (P < 0.001, OR 4.18 for resistance, 95%CI 2.06–8.54). In Table 4, risk factors for carriage of a resistant or non-susceptible strain are listed in full detail. Carriage of ciprofloxacin resistant strains was independent of antibiotic use.

**Table 4:** Risk factors for carriage of a resistant or a non-susceptible pneumococcal strain to penicillin and to erythromycin

	Univariate analysis			Multivariate analysis		
	
	OR	95%CI	*P*	OR	95%CI	*P*
	
Penicillin resistance						
Male gender	0.75	0.49–1.15	0.196	0.76	0.48–1.19	0.244
Age <3 years old	0.79	0.66–0.94	0.009	0.76	0.70–1.06	0.164
Sampling period*	0.48	0.31–0.73	0.001	0.57	0.36–0.92	0.023
Antibiotic use in the last month	1.94	1.26–2.98	0.002	2.00	1.26–3.17	0.003
Antibiotic use in the last trimester	1.07	0.70–1.63	0.749	1.09	0.69–1.71	0.696
History of acute otitis media episodes	1.07	0.70–1.63	0.749	0.90	0.54–1.51	0.709
History of respiratory tract infections	0.89	0.50–1.58	0.652	0.96	0.48–1.92	0.928
Other sibling(s) in the household <8 years old	0.96	0.62–1.47	0.814	1.07	0.67–1.71	0.751
Steroid use	1.02	0.64–1.59	0.971	1.20	0.715–2.02	0.486
Admission to the hospital in the last year	2.40	0.83–6.87	0.159	2.57	0.72–9.18	0.145
Prefecture of Thessaloniki**	0.42	0.19–0.9	0.026	0.33	0.13–0.83	0.019
Prefecture of Evia(Halkida)**	1.11	0.66–0.87	0.685	0.91	0.43–1.94	0.825
Prefecture of Evros(Alexandroupolis)**	0.57	0.17–1.92	0.458	0.52	0.14–1.91	0.328
Prefecture of Epirus(Ioannina)**	0.49	0.11–2.13	0.564	0.20	0.01–1.30	0.635
Prefecture of Peloponnese**	0.98	0.53–1.81	1.000	1.09	0.52–2.29	0.803
Prefecture of Herakleion, Crete**	2.17	1.11–4.22	0.036	1.69	0.70–4.07	0.238
**Erythromycin resistance**						
Male gender	1.06	0.78–1.45	0.693	1.00	0.72–1.39	0.958
Age <3 years old	0.82	0.72–0.94	0.005	0.866	0.74–1.00	0.061
Sampling period*	0.46	0.33–0.63	< 0.001	**0.52**	**0.37–0.73**	**< 0.001**
Antibiotic use in the last month	1.43	1.04–1.96	0.025	**1.92**	**1.35–2.72**	**< 0.001**
Antibiotic use in the last trimester	1.19	0.87–1.63	0.266	1.30	0.94–1.80	0.112
History of acute otitis media episodes	1.39	1.02–1.90	0.034	1.13	0.78–1.64	0.508
History of respiratory tract infections	0.78	0.50–1.20	0.262	**0.57**	**0.34–0.95**	**0.031**
Other sibling(s) in the household <8 years old	0.93	0.68–1.28	0.686	0.89	0.63–1.26	0.534
Steroid use	1.33	0.96–1.84	0.081	1.20	0.81–1.77	0.356
Admission to the hospital in the last year	0.77	0.27–2.19	0.801	0.87	0.23–3.23	0.846
Prefecture of Thessaloniki**	1.06	0.70–1.61	0.752	0.99	0.56–1.75	0.993
Prefecture of Evia(Halkida)**	1.29	0.87–1.89	0.194	1.03	0.59–1.78	0.913
Prefecture of Evros(Alexandroupolis)**	0.42	0.17–1.03	0.061	0.50	0.19–1.32	0.165
Prefecture of Epirus(Ioannina)**	1.28	0.57–2.86	0.531	1.39	0.51–3.80	0.516
Prefecture of Peloponnese**	0.36	0.36–0.97	0.017	**0.41**	**0.21–0.81**	**0.010**
Prefecture of Herakleion, Crete**	4.22	2.24–7.98	< 0.001	**3.26**	**1.55–6.84**	**0.002**

**Penicillin nonsusceptibility**						
Male gender	0.89	0.79–1.47	0.638	0.89	0.65–1.23	0.499
Age <3 years old	0.78	0.68–0.89	< 0.001	**0.82**	**0.71–0.96**	**0.013**
Sampling period*	0.5	0.37–0.69	< 0.001	**0.58**	**0.42–0.82**	**0.002**
Antibiotic use in the last month	1.71	1.23–2.38	0.001	**1.66**	**1.17–2.35**	**0.004**
Antibiotic use in the last trimester	1.36	1.00–1.84	0.045	**1.48**	**1.07–2.04**	**0.017**
History of acute otitis media episodes	1.04	0.77–1.41	0.815	0.82	0.56–1.18	0.297
History of respiratory tract infections	1.06	0.68–1.65	0.825	1.13	0.68–1.89	0.624
Other sibling(s) in the household <8 years old	0.84	0.61–1.28	0.686	0.85	0.61–1.20	0.379
Steroid use	1.30	0.95–1.79	0.102	1.36	0.93–2.01	0.108
Admission to the hospital in the last year	1.52	0.59–3.91	0.453	1.97	0.61–6.32	0.251
Prefecture of Thessaloniki**	0.89	0.59–1.35	0.678	0.48	0.27–0.86	**0.014**
Prefecture of Evia(Halkida)**	1.12	0.76–1.64	0.554	0.73	0.42–1.26	0.265
Prefecture of Evros(Alexandroupolis)**	0.21	0.074–0.63	0.005	0.19	0.06–0.57	**0.003**
Prefecture of Epirus(Ioannina)**	0.43	0.16–1.16	0.098	0.17	0.03–0.81	**0.026**
Prefecture of Peloponnese**	0.69	0.43–1.10	0.143	0.70	0.40–1.22	0.215
Prefecture of Herakleion, Crete**	1.91	1.05–3.48	0.003	1.39	0.68–2.62	0.357

**Erythromycin nonsusceptibility**						
Male gender	1.08	0.79–1.47	0.638	1.02	0.74–1.41	0.881
Age <3 years old	0.8	0.7–0.92	0.002	**0.84**	**0.72–0.97**	**0.023**
Sampling period*	0.46	0.34–0.64	< 0.001	**0.53**	**0.37–0.74**	**< 0.001**
Antibiotic use in the last month	1.81	1.3–2.52	< 0.001	**1.86**	**1.31–2.64**	**< 0.001**
Antibiotic use in the last trimester	1.44	1.05–1.97	0.021	1.32	0.95–1.82	0.091
History of acute otitis media episodes	1.44	1.06–1.96	0.019	1.16	0.80–1.67	0.426
History of respiratory tract infections	0.77	0.50–1.19	0.263	0.65	0.34–0.94	**0.028**
Other sibling(s) in the household <8 years old	0.97	0.71–1.33	0.873	0.92	0.65–1.30	0.662
Steroid use	1.26	0.91–1.73	0.610	1.13	0.77–1.67	0.519
Admission to the hospital in the last year	0.74	0.26–2.11	0.802	0.86	0.23–3.17	0.820
Prefecture of Thessaloniki**	1.02	0.67–1.54	0.916	0.92	0.52–1.62	0.794
Prefecture of Evia(Halkida)**	1.33	0.90–1.95	0.162	1.12	0.65–1.94	0.671
Prefecture of Evros(Alexandroupolis)**	0.40	0.16–0.99	0.042	0.46	0.17–1.21	0.120
Prefecture of Epirus(Ioannina)**	1.45	0.65–3.22	0.399	1.59	0.60–4.25	0.347
Prefecture of Peloponnese**	0.34	0.12–0.92	0.002	**0.38**	**0.2–0.75**	**0.005**
Prefecture of Herakleion, Crete**	4.02	2.13–7.58	< 0.001	**3.39**	**1.61–7.17**	**0.005**

* reference category for period of sampling: February

** reference category for prefecture of sampling: Athens, prefecture of Attika

### Use of antibiotics and resistance

A significant number of children from the total study population had at least one antibiotic course in the month preceding sampling (31.03%) or in the previous trimester (42.43%). Administration of antibiotics did not differ among geographic regions. The majority of antibiotic courses in the carriers' group were prescribed by a doctor either in the preceding month (92.5%) or trimester (87.8%). The rest of the consumed antibiotics were purchased over the counter by parents' initiative (5.55% and 9.8%) or by pharmacists' advice (1.85% and 2.3% of cases for the last month and trimester respectively).

The most common infections for prescribing an antibiotic were similar in rates in both groups (carriers and non-carriers). Rates for most common causes were as follows: acute otitis media 33.56 – 31%, tonsillitis 13.34 – 11%, lower respiratory tract infections 4.26 – 4%, and virus-related respiratory tract infections 28.2 – 28% (mostly rhinopharyngitis, tracheobronchitis and laryngitis), respectively for each group. Most commonly prescribed antibiotics were amoxicillin-clavulanate (35%) and oral 2^nd ^generation cephalosporins (31%), namely cefaclor, cefprozil and cefuroxime axetil.

### Serotype distribution

Among the 380 pneumococcal strains displaying resistance to at least 1 antibiotic class, serotyping was available for 317 strains (83.5% of resistant strains). The distribution of serotypes per geographic region and the respective coverage of the heptavalent pneumococcal conjugate vaccine is presented in Table 5. Serotypes 19F, 14, 9V, 23F and 6B formed 70.6% of the total number of resistant strains serotyped.

**Table 5:** Serotype distribution among pneumococcal strains displaying resistance to at least one antibiotic class (n = number of strains, % = percent of strains per geographic region).

	*Athens* *(n, %)*	*Halkida* *(n, %)*	*Thessaloniki* *(n, %)*	*Ioannina* *(n, %)*	*Alexandroupolis* *(n, %)*	*Herakleion* *(n, %)*	*Peloponnese* *(n, %)*	*Total* *(n,%)*
PCV7* serotypes	**80 (70.17)**	**42 (73.68)**	**33 (58.92)**	**10 (76.92)**	**6 (54.55)**	**25 (75.76)**	**30 (90.91)**	**226 (71.29)**
**19F**	32 (28.07)	20 (35.09)	9 (16,07)	2 (15.38)	3 (27.27)	11 (33.33)	10 (30.3)	87 (27.44)
**14**	10 (8.77)	6 (10.53)	10 (17.86)			11 (33.33)	3 (9.09)	40 (12.62)
**9V**	20 (17.54)		2 (3.57)	2 (15.38)			10 (30.3)	34 (10.73)
**23F**	10 (8.77)	6 (10.53)	6 (10.71)	5 (38.46)	1 (9.09)	1 (3.04)	4 (12.13)	33 (10.41)
**6B**	8 (7.02)	10 (17.54)	5 (8.93)	1 (7.69)	2 (18.18)	2 (6.06)	2 (6.06)	30 (9.46)
**18C**			1 (1.79)				1 (3.03)	2 (0.63)

**PCV7-related serotypes**	**17 (14.92)**	**10 (17.55)**	**11 (19.66)**	**0**	**2 (18.18)**	**4 (12.12)**	**0**	**43 (13.56)**
**6A**	7 (6.14)	3 (5.26)	5 (8.93)		0	2 (6.06)		17 (5.36)
**Other related**	10 (8.78)	7 (12.29)	6 (10.73)		2 (18.18)	2 (6.06)		26 (8,20))

**Non PCV7 serogroups**	**10 (8.77)**	**3 (5.26)**	**8 (14.28)**	**3 (23.08)**	**1 (9.09)**	**2 (6.06)**	**3 (9.09)**	**32 (10.1)**

**Nontypeable strains**	**7 (6.14)**	**2 (3.51)**	**4 (7.14)**	**0**	**2 (18.18)**	**2 (6.06)**	**0**	**16 (5.05)**

**Total**	114 (100)	57 (100)	56 (100)	13 (100)	11 (100)	33 (100)	33 (100)	317 (100)

*PCV7: heptavalent pneumococcal conjugate vaccine

## Discussion

The present study is the first surveillance study conducted in Greece at nation-wide level in the pre-vaccination era, with representative samples per geographical region, according to the age-specific population distribution in the most recent records of the National Registry in March 2001 [12].

Carriage rates in the present study varied significantly with age, period of sampling and the geographic area visited. This significant trend differentiation stresses the importance of sequential sampling in a specific area and multifocal sampling at a specific time-point, as useful tools in the epidemiological studies of resistance. It must be noted that regional differences in carriage rates did not affect resistance/nonsusceptibility rates. Resistance rates differed among different regions. This difference per region renders the results of a multicenter study even more valuable, as it may predict similar trends in the clinical isolates [13]. In the present study serotyping was performed only in the resistant strain population. Thus, the association of specific serotypes with specific resistance patterns cannot be accurately performed and regional variability in resistance rates for penicillin and erythromycin in relation to serotype distribution requires caution.

One of the risk factors amenable to intervention for carrying a resistant or a non-susceptible pneumococcal strain in the nasopharynx for a healthy preschool child was the use of antibiotics in the month preceding sampling. Additionally, antibiotic use was related to the isolation of combined penicillin-erythromycin resistant, as well as multiresistant pneumococcal strains Numerous studies [14-16] have pointed out antibiotic consumption as a very strong pressure factor for the development of resistance, irrespective of ecological setting (individuals-community). Chung *et al *[17] have recently shown that prescription of amoxicillin doubled the risk of recovery of β lactamase producing *Haemophilus influenzae *strains. Although this effect was transitory at an individual level (2 weeks), it was sufficient to sustain a high level of antibiotic resistance in the study population.

Acute otitis media was the most frequent infection for which a child received antibiotics in our study. Nonetheless, a similar proportion of children (approx. 30%) consumed antibiotics for infections which seemed to be of viral cause by all descriptions in the questionnaire used. It must be stressed that acute otitis media does not always require prompt administration of antibiotics, as it may be of viral origin in certain cases. In Finland Palmu *et al *recorded negative cultures of middle ear fluid in approximately 35% of children with acute otitis media in 2004 [18]. Moreover, a wait-and-see approach to acute otitis media treatment in the paediatric population seems to reduce antibiotic consumption and subsequent resistance without jeopardizing children's safety and well-being [19].

Approximately 90% of the prescriptions originated from practicing physicians in the community, while in the remaining 10% of cases, parents had access to antibiotics without doctor's prescription, although this is not abiding by antibiotics policy law [20]. As demonstrated by Guillemot *et al *[21], intensive educational strategies, targeting to optimize antibiotic use both in terms of indications and of dose/duration of administration, significantly reduced the colonization with penicillin non-susceptible pneumococci in areas with high prevalence of resistance.

The heptavalent pneumococcal vaccination (PCV7) covered 71.32% of resistant strains in total in the present study. The most prevalent serotypes in our population carried the heavier burden of antibiotic resistance. Implementation of the heptavalent vaccine has been shown to be highly efficacious in decreasing the incidence of vaccine-type antibiotic-resistant strains in disease and colonization [5]. A study by Grivea *et al*[8], at a regional level in Thessaly, Central Greece between 2005–2007, showed that increasing rates of PCV7 population coverage was associated with a reduction in colonization by PCV7-related serotypes in vaccinated (from 33% to 8.6%, *P *< 0.001) and unvaccinated children (from 36% to 17%, *P *= 0.05). Moreover, a decrease in the rate of highly penicillin resistant pneumococcal isolates was recorded only in vaccinated children (from 11% to 0.6%, *P *= 0.001), whereas rates for penicillin intermediate strains were not affected.

In a community setting of high antibiotic consumption, Frazao *et al*[22] showed that PCV7 vaccination was successful in the reduction of vaccine related strains, but it did not change the frequency of nasopharyngeal carriage of drug resistant pneumococci, implying the crucial role of antibiotic consumption on the serotype replacement phenomenon by drug-resistant non-vaccine related pneumococcal strains.

To reduce antibiotic resistance in the community, reducing unnecessary antibiotic prescriptions combined with the implementation of the heptavalent conjugate pneumococcal vaccine are probably two of the most efficient strategies. In a large scale prospective study in France from 2001 to 2005 in children presenting with acute otitis media, Cohen *et al *[23] showed that unvaccinated children who had received antibiotics within the last three months had a 4-fold greater risk of carrying a penicillin resistant strain than vaccinated children who had not received antibiotics. Moreover, vaccination reduced the antibiotic consumption from 51.6% to 38.5%, as well as the risk for a child to carry a penicillin resistant pneumococcal strain from 15% to 3%.

## Conclusion

Colonization with a pneumococcal strain is a usual event in the everyday life of preschool children. Epidemiological data derived from the present surveillance study stress the importance of recent antibiotic use in the colonization of otherwise healthy children's nasopharynx by resistant strains of *S pneumoniae*. The heptavalent pneumococcal conjugate vaccine, as shown from the serotype distribution in the pre-vaccination era, could provide coverage against the majority of resistant strains. In settings with a high antibiotic consumption profile in the community, like the one described in the present study, prudent use of antibiotics at an individual level coupled with vaccination could prove helpful in decreasing pneumococcal resistance rates in the community, thus facilitating treatment of pneumococcal infection.

## Competing interests

The study was financially supported by Bayer Hellas. Part of serotype antisera was granted by Sanofi-Aventis, Hellas.

## Authors' contributions

IK, GP, HG and KK designed the study, formed the study protocol, coordinated the participating centers, and drafted the manuscript. IK, GP and IM also participated in the collection of samples, questionnaire analysis and in vitro processing of samples. AA was responsible for the statistical analysis. RV participated in the vitro procedures of the study. ER was responsible for Thessaloniki participating center and helped to draft the manuscript. CA participated in the collection of samples and questionnaire analysis in Thessaloniki. DAK, VS, GLD, GS, CK and IP formed and coordinated local teams responsible for the collection of samples and questionnaire analysis. All authors read and approved the final manuscript.

## Pre-publication history

The pre-publication history for this paper can be accessed here:

http://www.biomedcentral.com/1471-2334/9/120/prepub
